# New insight into the role of phosphodiesterase 3A in porcine oocyte maturation

**DOI:** 10.1186/1471-213X-6-47

**Published:** 2006-10-12

**Authors:** Maxime Sasseville, Nancy Côté, Christine Guillemette, François J Richard

**Affiliations:** 1 Centre de Recherche en Biologie de la Reproduction, Département des Sciences Animales, Université Laval, Québec, Canada

## Abstract

**Background:**

The ovulatory surge of gonadotropins triggers oocyte maturation and rupture of the ovarian follicle. The resumption of nuclear maturation in the oocyte from the prophase stage is characterized by germinal vesicle breakdown (GVBD). It has previously been shown that specific inhibition of cAMP degradation by PDE3 prevents the resumption of oocyte meiosis. However, no report has characterized the activity of PDE3 in the porcine oocyte, or the implication of the cAMP-PDE3 pathway in the entire nuclear maturation process. In this study, PDE3 activity in the oocyte was assessed during *in vitro *maturation (IVM) and the possible roles of the cAMP-PDE3 pathway in the resumption and progression of meiosis were investigated in terms of different models of oocyte maturation.

**Results:**

Cyclic AMP-degrading PDE activity was detected in the cumulus-oocyte complex (COC) and was partially inhibited by a specific PDE3 inhibitor, cilostamide. When measured only in the denuded oocyte, PDE activity was almost completely inhibited by cilostamide, suggesting that cAMP-PDE3 activity is the major cAMP-PDE in porcine oocytes. PDE3A mRNA was detected by RT-PCR. PDE3 activity did not vary significantly during the early hours of IVM, but a maximum was observed at 13 hours. In cumulus-oocyte complexes, meiosis resumed after 20.81 hours of culture. PDE3 inhibition no longer maintained meiotic arrest if sustained beyond 17.65 hours of IVM, 3 hours prior to resumption of meiosis. Thereafter, PDE3 inhibition progressively lost its efficacy in GVBD. When the protein phosphatase 1 and 2A inhibitor okadaic acid was continuously or transiently (3 hours) present during IVM, meiosis resumed prematurely; PDE3 inhibition was unable to prevent GVBD. However, PDE3 inhibition in COC treated with OA for 3 hours significantly delayed meiosis at the intermediate stage.

**Conclusion:**

The present investigation has demonstrated that PDE3A is the major cAMP-degrading PDE in the oocyte. It regulates the resumption of meiosis until 3 hours prior to GVBD and transiently affects meiotic progression.

## Background

Oogonia entering meiosis progress to the diplotene stage of the first meiotic prophase, at which they display a characteristic nucleus commonly known as the germinal vesicle (GV). Oocytes arrested at the GV stage, prophase I of meiosis, enter a growth phase during which the follicle differentiates from the primary to the preovulatory stage. Oocytes acquire the capacity to resume meiosis during this period and rely on 3'5'-cyclic adenosine monophosphate (cAMP) to prevent premature resumption. Cyclic AMP is produced in the somatic granulosa cells and transferred through gap-junction channels [1]. Recent findings have shown that cAMP-producing capacity is also important in maintaining the prophase I-arrested state in oocytes [2]. Cyclic AMP activates the cAMP-dependent protein kinase (PKA) by binding to the two regulatory subunits, liberating the two active catalytic subunits. High concentrations of cAMP and correspondingly high PKA activity within the oocyte prevent the resumption of meiosis, as shown using cAMP analogues [3,4], adenylyl cyclase activators [5,6] and invasive adenylyl cyclase treatments [7]. Microinjection of Xenopus oocytes with the catalytic subunit of PKA also maintains them in the GV stage [8]. However, the PKA-mediated mechanism that maintains meiotic arrest is not fully understood, although interesting advances have recently been made on possible PKA targets in mouse oocytes, such as CDC25B phosphatase and Wee1B kinase, two cell cycle regulators [9].

Cyclic AMP is degraded by members of the phosphodiesterase (PDE) family [10]. PDEs are important regulators of ovarian physiology. During the past few years, PDE3 family-specific inhibitors (e.g. cilostamide, milrinone, Org 9935) have been shown to block the resumption of meiosis efficiently in murine [11], rat [12], bovine [13,14], porcine [15,16], macaque [17] and human [18] oocytes. PDE3A- and PDE4D-null female mice respectively show infertility and impaired fertility [19,20]. Interestingly, PDE3A-deficient mice ovulate fully-grown oocytes, but they fail to resume meiosis [20]. In rat oocytes, a cAMP-degrading activity sensitive to cilostamide (PDE3-specific) is increased 2-fold prior to resumption of meiosis, strongly suggesting that cAMP degradation is actively regulated in the oocyte and that regulation of PDE3A is part of the mechanism controlling resumption of meiosis [21].

Nuclear maturation in the oocyte and rupture of the follicle are triggered by an ovulatory surge of gonadotropins *in vivo*. The latter is characterized by breakdown of the germinal vesicle (GVBD). GVBD is correlated with an increased activity of M phase-promoting factor (MPF). MPF is essential for GVBD, chromosome condensation, formation of microtubules around the condensed chromosomes and their organization into a bipolar structure [22]. The complete molecular pathways linking the endocrine cues to the first activation of MPF in the oocyte during nuclear maturation are unclear. However, it is known that gonadotropins trigger multiple phosphorylation cascades and second messenger signalling pathways in the somatic compartment of the ovarian follicle, and these are critical for the correct timing of oocyte maturation. Simultaneously with activation of the MPF, mitogen-activated protein kinase (MAPK) activates phosphorylation in the oocyte and is believed to be implicated in chromosome condensation and microtubule organisation [23]. After its initial increase, MPF activity stays high until metaphase I before decreasing prior to homologous chromosome segregation and extrusion of the first polar body [22]. A second increase in MPF activity maintains metaphase II prior to sperm penetration. Although this second activation is known to depend on factors such as cyclin B synthesis, presence of the nucleus and MAPK activity, the precise implication of the PDE3A/cAMP/PKA pathway remains unknown [22,24]. Our laboratory has recently described physiological evidence showing the pivotal role of cAMP and its modulation by PDE3 in regulating the resumption of meiosis in porcine oocytes [15]. Since no study has reported the progression of PDE3 activity during porcine oocyte nuclear maturation, the goal of the present study was to measure PDE3 activity in the oocyte during *in vitro *maturation (IVM) and to characterize the effect of PDE3 inhibition on different steps of nuclear maturation.

## Results

### Contributions of different cAMP-degrading PDEs in the oocyte

**Figure 1:**
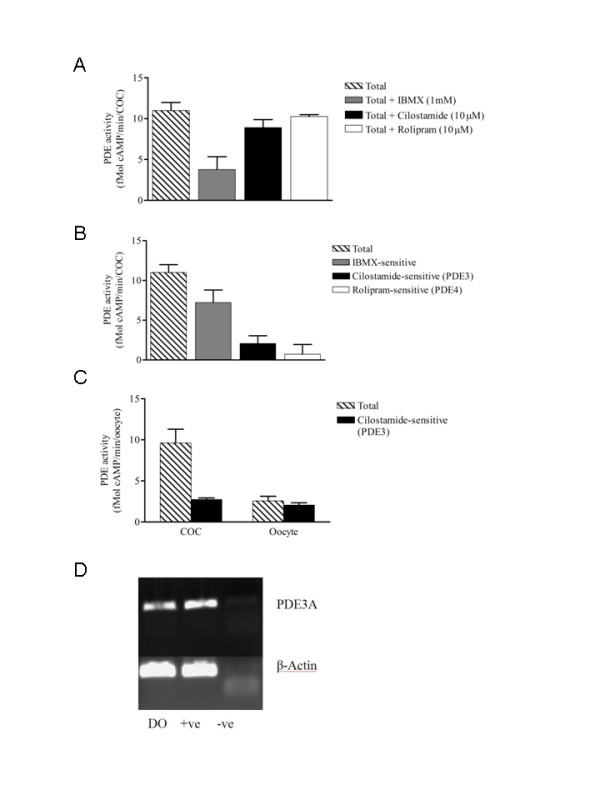
**Cyclic AMP-degrading PDE activity in the oocyte**. A. Cyclic AMP-PDE activity was measured in the COC either alone or in the presence of a non-specific PDE inhibitor (IBMX, 1 mM) or of PDE3 (cilostamide, 10 μM) or PDE4 (rolipram, 10 μM) specific inhibitors. B. Total and calculated IBMX-sensitive, cilostamide-sensitive (PDE3) and rolipram-sensitive (PDE4) PDE activities in the COC are presented. To determine the inhibitor-sensitive contributions to the overall PDE activity, the activity measured in the presence of the respective inhibitors was subtracted from the total PDE activity. C. Comparison of the total (hatched bars) and cilostamide-sensitive (PDE3) (closed bars) PDE activities in the COC or DO after recovery from the ovary. A total of 1290 oocytes were used. Each column represents the mean ± SEM of at least three replicates. D. Reverse transcription-PCR analysis of β-actin and PDE3A mRNA in porcine oocyte cDNA. Ethidium bromide-stained 1% agarose gels were used to resolve the amplified products. DO: denuded oocyte cDNA; +ve: positive control; -ve: negative control without cDNA. A PDE3A amplicon in the oocytes was sequenced and found to have 98.3% identity with the published sequence. Representative pictures of the three different experiments are shown.

The PDE assay was optimized for porcine COC. Increasing numbers of COC were used to measure cAMP hydrolysis. The hydrolytic activity was linearly related to the number of COC and 10 COC or denuded oocytes were used (data not shown). In experiment 1, cAMP-degrading PDE3 activity was measured in cumulus-oocyte complexes (COC) after recovery from the ovary with or without PDE inhibitors. The total cAMP-PDE activity in porcine COC was 10.98 ± 0.99 fmol cAMP degraded/min/COC, and this activity was decreased by non-specific and family-specific PDE inhibitors. Figure 1A shows that 66% of the cAMP-degrading PDE activity is inhibited by the non-specific inhibitor 3-isobutyl-methylxanthine (IBMX), while 19% and 7% inhibition are achieved, respectively, by PDE3-specific (cilostamide) and PDE4-specific (rolipram) inhibitors (figure 1A). IBMX inhibits every cAMP-PDE subtype except for PDE8. The IBMX-sensitive (PDE1, 3, 4, 7, 10 and 11), cilostamide-sensitive (PDE3) and rolipram-sensitive (PDE4) activities were calculated as described in the experimental design section and reported in figure 1B. The cAMP-degrading PDE activity in the oocyte was 2.57 ± 0.57 fmol cAMP degraded/min/oocyte (figure 1C). Cyclic AMP-PDE3 activity measured in the denuded oocyte was almost equivalent to the total activity. Moreover, these two activities are similar to the cAMP-PDE3 specific activity measured in COC (figure 1C), suggesting that PDE3 is the predominant PDE isoform in porcine oocytes. PDE3A mRNA was detected in the oocyte by RT-PCR (figure 1D). To confirm the identity of the amplification products further, they were sequenced and found to be 98.3%, 89.7%, 81.4% and 80.9% identical to published porcine, human, mouse and rat sequences, respectively.

**Figure 2:**
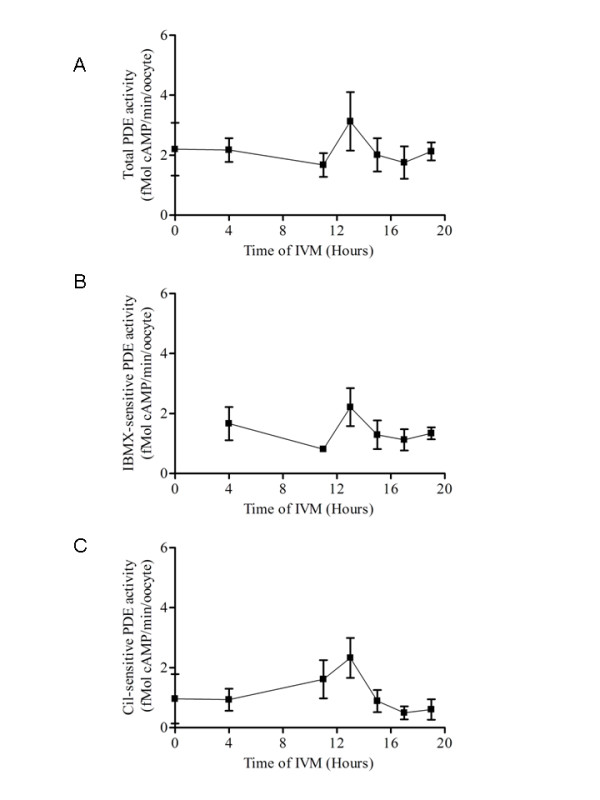
**Cyclic AMP-degrading PDE activity measured in the oocyte during IVM**. Cyclic AMP-PDE activity was measured in isolated denuded oocytes (stripped of cumulus cells) at different time points during COC-cultured IVM. Total (A), IBMX-sensitive (B) and cilostamide-sensitive (C) cAMP-PDE activities measured in the oocyte during COC-cultured IVM. A total of 2100 oocytes were used. Each activity value represents the mean ± SEM of at least three replicates. Statistical analysis by ANOVA showed no significant variation in PDE activities during the IVM period (P > 0.05).

In experiment 2, oocytes were cultured as COC. At the end of the culture, the cumulus cells were removed by gentle pipetting and after several washes the oocytes were assessed for cAMP-degrading activity. PDE3 activity in the oocyte was measured at different time points during IVM. No statistically significant effect of either total, IBMX-sensitive or cilostamide-sensitive (PDE3) phosphodiesterase activities was observed during IVM (figure 2). However, maximum PDE activity was measured at 13 hours of IVM, after which the activity returned to basal level.

### Time-dependent ability of specific PDE3 inhibition to maintain COC in meiotic arrest

**Figure 3:**
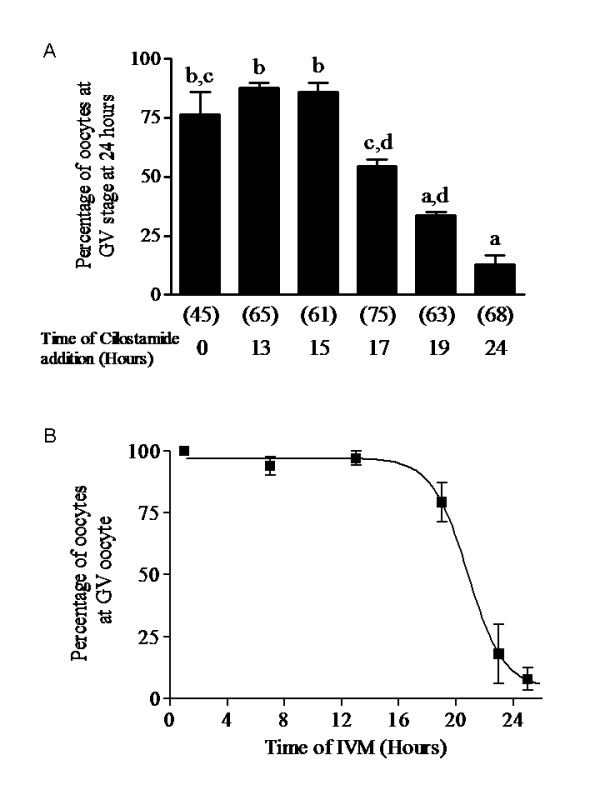
**Time-dependent ability of specific PDE3 inhibitionto maintain COC in meiotic arrest**. (A) COC were cultured in cilostamide-free medium for different times followed by the addition of a PDE3 inhibitor, cilostamide (10 μM), up to 24 hours of IVM. Each column represents the mean percentage of GV stage oocyte at 24 hours ± SEM of at least three replicates. The number of oocytes per treatment is indicated under each bar. Statistical analysis by ANOVA showed that the treatments had significant effects (P < 0.0001). Different letters indicate significant differences (P < 0.05) according to the Bonferroni *post hoc *test. (B) Time-course of resumption of meiosis in porcine COC over 25 hours. Non-linear regression showed that 50% of the oocytes had resumed meiosis at 20.8 hours of culture. For each time point, 60 oocytes were used. Each value represents the mean percentage of GV stage oocytes ± SEM of at least three replicates.

The efficacy of the PDE3 inhibitor, cilostamide, in controlling GVBD was tested. In the previous experiments, specific PDE3 inhibitors were added at the beginning of IVM [15,16]. In experiment 3, the addition of cilostamide was postponed for several hours after the beginning of IVM. The inhibitor was still efficient at controlling GVBD when added after 13 and 15 hours of IVM (figure 3A). When PDE3 inhibition with cilostamide was still further delayed there was a gradual decrease in its control of GVBD (figure 3A). A linear regression calculation showed that 50% of the COC remain in GV when PDE3 is inhibited at 17.65 hours, which is approximately 3 hours prior to GVBD (figure 3B) and 5 hours after the peak of PDE activity in the oocyte (figure 2C). As shown in figure 3B, most of the oocytes resumed meiosis normally under our culture conditions within a narrow window of 4 hours between 19 and 23 hours of IVM (regression analysis, 20.81 hours of IVM for 50% GVBD). This result suggests that the cAMP-PDE3 pathway actively controls maintenance of the GV stage during at least the first 15 hours of IVM. After 19 hours of cilostamide-free culture conditions, the addition of cilostamide for the last 4 hours had no significant effect on maintaining the oocytes in the GV stage (figure 3A). In addition, maximum PDE3 activity occurs at approximately 13 hours of IVM (figure 2), which is 6 hours prior to the end of period in which PDE3 inhibition can prevent GVBD. At 19 hours, cAMP-PDE3 activity is minimal (figure 2C), consistent with its inefficacy in controlling GVBD.

**Figure 4:**
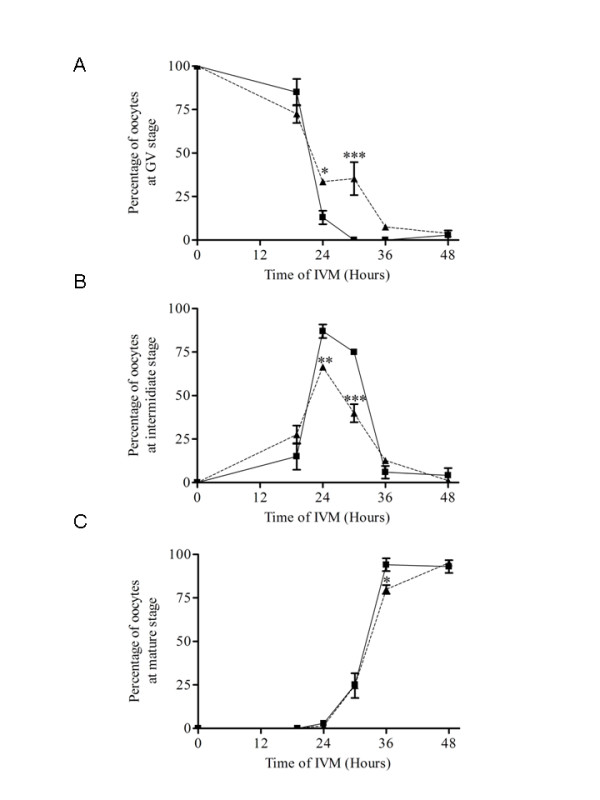
**Effect of specific PDE3 inhibitor, cilostamide, on oocyte maturation**. COC were cultured first in cilostamide-free IVM medium for 19 hours followed by the addition of cilostamide (20 μM) for up to 48 hours in culture. Control (--■--) and cilostamide-treated (---▲---) experiments show the percentages of oocytes in GV arrest (A), intermediate stage (B) and mature stage (C). Values represent the means ± SEM of at least three replicates. A total of 590 oocytes were used. Statistical analysis by ANOVA showed that effects of treatments were significant (P = 0.001). Asterisks indicate significant differences according to the Bonferroni *post hoc *test (*, P < 0.05; **, P < 0.01; ***, P < 0.001).

MPF activation is critical for the resumption of oocyte meiosis. As proposed above, the period preceding GVBD is cAMP-PDE3-sensitive. Since MPF activity during IVM in vertebrate oocytes changes biphasically before reaching the second meiotic arrest in metaphase II, experiment 4 was designed to verify whether the second MPF activation depends on cAMP-PDE3 activity. PDE3 was inhibited with cilostamide after 19 hours of IVM and meiotic maturation was assessed immediately and at 24, 30, 36 and 48 hours afterwards. Of the cilostamide-treated COC, 66.5% resumed meiosis during 19 and 24 hours of IVM (the cilostamide-insensitive group) while 33.5% remained in GV arrest (the cilostamide-sensitive group; figure 4A). The cilostamide-insensitive oocytes reached an intermediate nuclear stage at 24 hours (figure 4B). Of this group, 40% reached the mature nuclear stage at 30 hours, equivalent to the control oocytes (figure 4C). This result suggests that oocytes that resume meiosis despite PDE3 inhibition, the cilostamide-insensitive group, reach the mature nuclear stage at a similar rate to their control counterparts and therefore are not affected by PDE3 inhibition. The significantly higher percentages of oocytes in GV arrest at 24 (P < 0.05) and 30 (P < 0.001) hours compared to control oocytes reveal that cilostamide treatment transiently maintained the cilostamide-sensitive oocytes in meiotic arrest (figure 4A). The significantly lower percentage of oocytes at an intermediate nuclear stage at 24 (P < 0.01) and 30 (P < 0.001) hours is the result of the delay in meiotic resumption for 30% of the oocytes (figure 4A and 4B). The transition of oocytes from intermediate to mature stage at 24 and 30 hours is not compromised, since similar percentages of treated and untreated oocytes reach the mature stage at 30 hours (figure 4C). The delay in resumption of meiosis caused by cilostamide treatment can still be observed at 36 hours in the percentage of oocytes at the mature nuclear stage (79.9 ± 2.5% vs 94.1 ± 3.6%, cilostamide-treated vs control, figure 4C). However, oocytes under both treatments have completed meiotic maturation by 48 hours, showing that the effect of late PDE3-specific inhibition on resumption and progression of meiosis is transient and fully reversible. These results suggest that PDE3 inhibition affects GVBD under normal IVM conditions, but without affecting the progression of meiosis after its resumption until metaphase II arrest.

### Effects of short OA exposure in the presence of PDE3 inhibition on oocyte nuclear maturation

**Figure 5:**
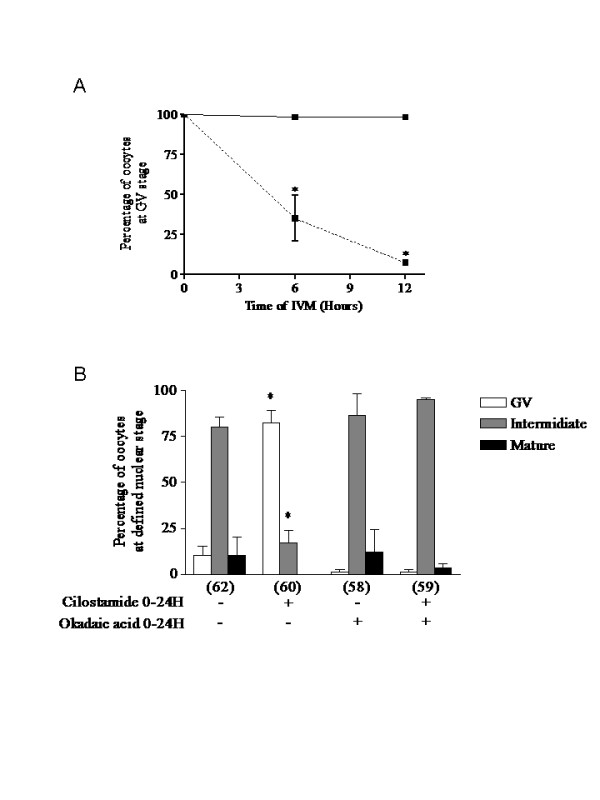
**Effect of specific inhibition of protein phosphatase 1/2A by okadaic acid on oocyte maturation**. A. COC were cultured in the presence of a protein phosphatase 1/2A inhibitor, okadaic acid (2 μM), for 6 or 12 hours of IVM and nuclear status was assessed as described in the Materials and Methods section. Results from COC cultured under control conditions (--■--) or treated with okadaic acid (---■---) are presented as the mean percentages of GV stage oocytes ± SEM of at least three replicates. A total of 217 oocytes were used. Statistical analysis by ANOVA showed that the treatments had significant effects (P < 0.0001). Asterisks indicate significant differences (P < 0.001) from the COC control according to the Bonferroni *post hoc *test. B. COC were cultured in the presence of okadaic acid (2 μM) and the specific PDE3 inhibitor cilostamide (20 μM) for 24 hours of IVM and were analysed to assess nuclear status. Results are given as the mean percentages of oocytes in GV arrest (open bars), intermediate stage (grey bars) and mature stage (closed bars) ± SEM of at least three replicates. The number of oocytes per treatment is indicated under each bar. Statistical analysis by ANOVA showed that the treatments had significant effects (P < 0.001). Asterisks indicate significant differences (P < 0.001) from control according to the Bonferroni *post hoc *test.

The protein phosphatase 1/2A (PP1/2A)-specific inhibitor okadaic acid (OA) has previously been used to stimulate chromatin condensation and premature GVBD in vertebrate oocytes [25]. In experiment 5, oocytes treated continuously with OA (2 μM) displayed significantly lower levels of arrest than control oocytes (P < 0.0001) at 6 and 12 hours of IVM (figure 5A). To study the effect of PDE3-specific inhibition on premature GVBD in OA-treated oocytes, oocytes were exposed to both cilostamide and OA for 24 hours of IVM. As reported previously, PDE3 inhibition by cilostamide prevents GVBD under normal IVM conditions (figure 5B) [15]. However, PDE3 inhibition no longer prevented GVBD in continuously OA-exposed oocytes (figure 5B). Even when OA addition was delayed for 6 hours, PDE3 inhibition did not prevent GVBD (data not shown). These results clearly suggest that inhibition of PP1/2A with OA affects the GVBD signalling cascade downstream of cAMP-PDE3A in pig oocytes.

**Figure 6:**
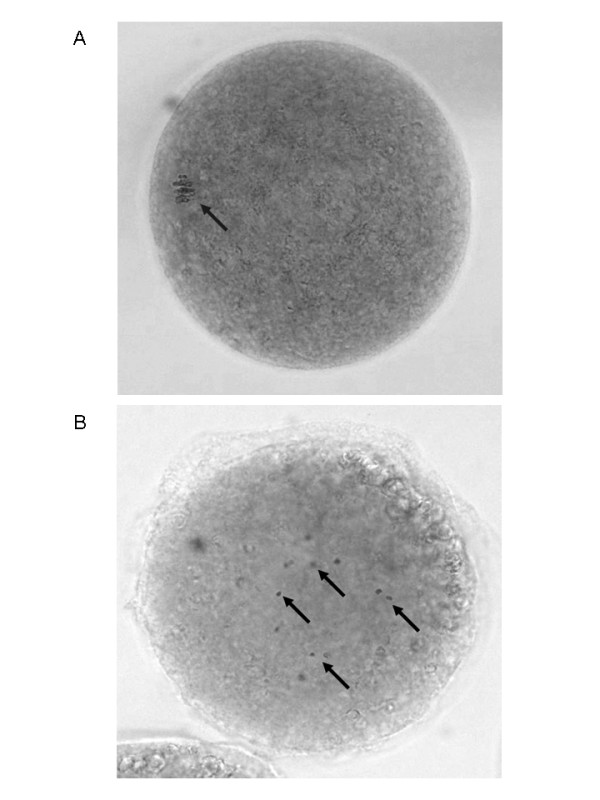
**Effects of protein phosphatase 1/2A inhibition on oocyte chromatin condensation**. (A) Photograph of an oocyte stained with aceto-orcein displaying the metaphase I chromatin configuration after 24 hours of IVM. (B) OA-treated oocyte (2 μM) after 24 hours of IVM displaying condensed bivalent chromatin that is scattered in the cytoplasm. Pictures were acquired on a Nikon Eclipse E600 with a Qimaging Retiga 1300 camera. The original magnification was X400.

**Figure 7:**
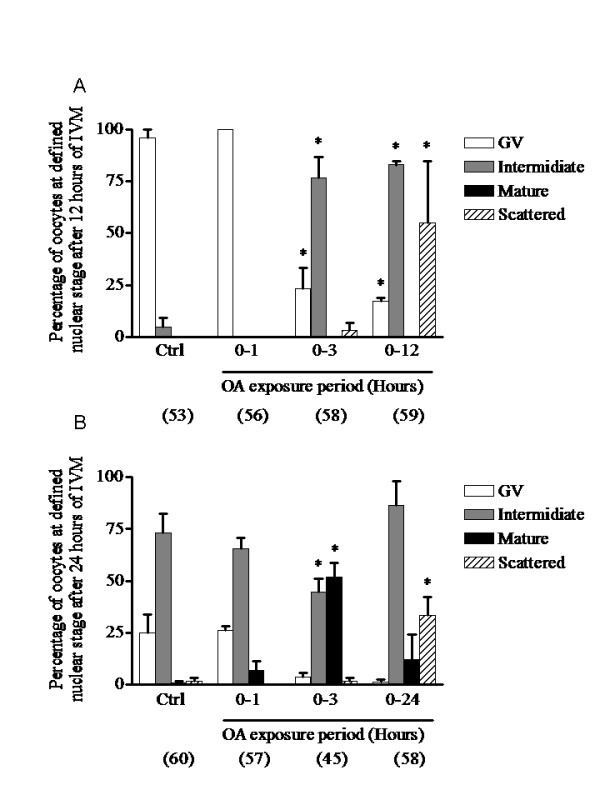
**Time-dependent effect of protein phosphatase 1/2A inhibition with okadaic acid on oocyte maturation**. COC were cultured in okadaic acid-supplemented medium for different times followed by culture in inhibitor-free medium for up to a total of 12 (A) or 24 (B) hours of IVM; nuclear status was then assessed. Each column represents the mean percentages of oocytes in GV arrest (open bars), intermediate stage (grey bars) and mature stage (closed bars) ± SEM of at least three replicates. The mean percentage of oocytes that displayed condensed scattered chromatin is presented (hatched bars) and is included in the intermediate stage percentage. The number of oocytes per treatment is indicated under each bar. Statistical analysis by ANOVA showed that the treatments had significant effects (P < 0.0001). Asterisks indicate significant differences (P < 0.01) from control according to the Bonferroni *post hoc *test.

Because continuous OA exposure causes meiotic spindle assembly defects and disperses the condensed chromosome configuration at the intermediate nuclear stage (figure 6B) instead of the normal metaphase I configuration (figure 6A) [23], a transient exposure of oocytes to OA was used in experiment 6. As shown in figure 6A, comparable intermediate nuclear stages could be observed at 12 hours in the continuously OA-treated and in the 3 hours OA-treated oocytes, while in the 1 hour OA-treated group the oocytes were still at the GV stage, as in the control group. Moreover, the 3 hours OA-treated group had a minimal percentage of oocytes with dispersed bivalent chromatin compared with the group continuously exposed for 12 hours (figure 7A). When assessed after 24 hours of IVM, the control and one hour OA-treated groups both displayed a high percentage of intermediate nuclear maturation stage oocytes (figure 7B). Meiosis did not progress significantly between 12 and 24 hours of IVM in continuously OA-exposed oocytes and still displayed a high percentage at the intermediate nuclear stage with a disperse chromatin configuration (figure 7B). Oocytes exposed for 3 hours to OA displayed 51.9 ± 6.7% of oocytes in the mature stage, indicating that they may progress through the intermediate stage. Considering these results, the next experiments were undertaken using transient (3 hours) exposure to OA.

**Figure 8:**
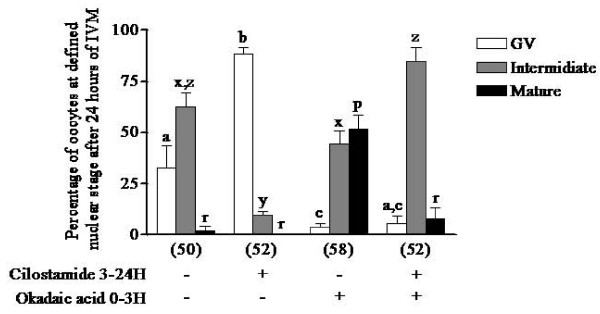
**Effects of specific PDE3 inhibition on maturation of OA-treated oocytes**. COC were cultured in okadaic acid (2 μM)-supplemented medium for 3 hours followed by culture in either inhibitor-free or cilostamide (20 μM)-supplemented medium for up to 24 hours of IVM. The nuclear status of the oocytes was then assessed. Each column represents the mean percentages of oocytes in GV arrest (open bars), intermediate stage (grey bars) and mature stage (closed bars) ± SEM of at least three replicates. The number of oocytes per treatment is indicated under each bar. Statistical analysis by ANOVA showed that the treatments had significant effects (P < 0.0001). Different letters indicate significant differences (P < 0.05) according to the Bonferroni *post hoc *test.

Experiment 7 was designed to determine the effect of PDE3 inhibition on 3 hours OA-treated oocytes. As with continuous OA treatment (figure 5B), PDE3 inhibition no longer prevented GVBD in transiently OA-treated oocytes (open bars in figure 8). These results also show that COC treated with OA for 3 hours have a higher percentage of mature stage oocytes at 24 hours (38.8% vs 1.1% of mature stage oocytes, OA-treated vs control, figure 8). PDE3 inhibition in 3 hours OA-treated COC affected meiosis by maintaining oocytes at the intermediate nuclear maturation stage (figure 8). These results suggest that inhibition of cAMP-PDE3 slows the progression of meiosis to the mature stage, consistent with a role in MPF activation.

**Figure 9:**
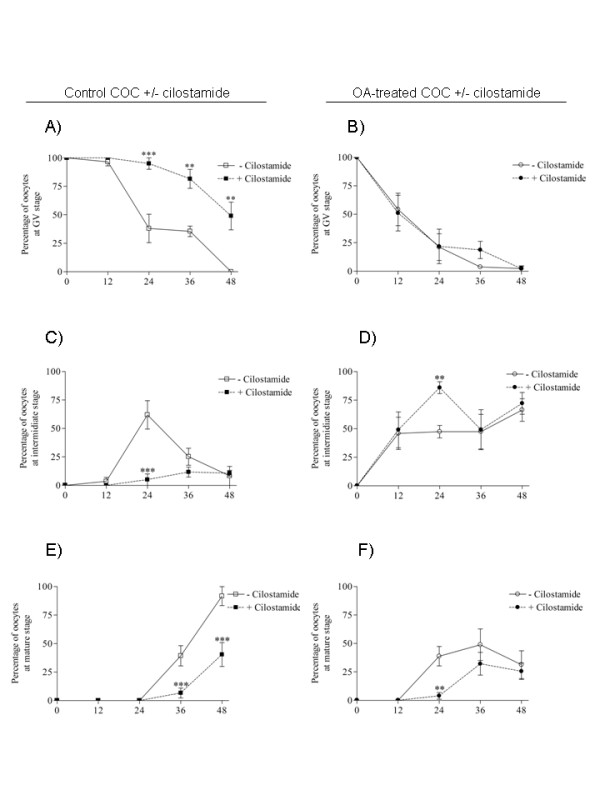
**Effects of specific PDE3 inhibition on maturation of OA-treated oocytes over 48 hours of IVM**. COC were initially cultured either in control conditions (A-C-E) or in okadaic acid (2 μM)-supplemented medium for 3 hours (B-D-F) Each group was cultured up to 12, 24, 36 and 48 hours of IVM alone (open symbols) or with cilostamide (20 μM, closed symbols). The nuclear status was then assessed in a sample of oocytes from each condition every 12 hours of IVM up to 48 hours. Results are presented as the mean percentages of oocytes in GV arrest (A-B), intermediate stage (C-D) and mature stage (E-F) ± SEM of at least three replicates. A total of 845 oocytes were used. Asterisks indicate significant differences between control and cilostamide treated-oocytes or between okadaic acid and okadaic acid-cilostamide-treated oocytes according to the Bonferroni *post hoc *test. (*, P < 0.05; **, P < 0.01; ***, P < 0.001).

In experiment 8, to understand the preceding findings further, the nuclear stage was assessed every 12 hours up to 48 hours of IVM after the treatments described above. PDE3 inhibition of COC caused a significantly higher percentage of GV stage oocytes after 24 (P < 0.001), 36 (P < 0.01) and 48 (P < 0.01) hours of IVM than in controls (figure 9A). It also prevented the increase of intermediate (24 hours) and mature (36 and 48 hours) stage oocytes (figure 9C, 9E). A transient OA exposure (3 hours) triggered a rapid loss of GV stage oocytes after 12 hours of IVM (figure 9A, 9B) and a significantly higher rate of mature stage oocytes after 24 hours of IVM compared to the control group (figure 9E, 9F). PDE3 inhibition of 3 hours OA-treated COC did not affect the decline in GV stage oocyte percentage after 12 or 24 hours of IVM (figure 9B). Consistent with results in figure 8, PDE3 inhibition of 3 hours OA-treated oocytes allowed intermediate stage oocytes to accumulate and the appearance of mature stage oocytes to be delayed after 24 hours of IVM in comparison to OA-treated oocytes (figure 9D, 9F). These results suggest that inhibition of cAMP-PDE3 transiently affects the progression of meiosis in 3 hours OA-treated oocytes.

## Discussion

This study demonstrates that in porcine oocytes, PDE3A is the major active cAMP-PDE; it efficiently regulates the resumption of meiosis until 3 hours before GVBD and transiently affects meiotic progression. Four lines of evidence support this conclusion: (1) cilostamide-sensitive cAMP-PDE activity in the oocyte represents the main cAMP-PDE activity; (2) PDE3A mRNA was detected in oocyte cDNA by RT-PCR; (3) PDE3 inhibition after 15 hours of IVM still prevents the resumption of meiosis; (4) PDE3 inhibition delays nuclear maturation in OA-treated oocytes.

Total and cilostamide-sensitive (PDE3) PDE activities in porcine COC and denuded oocytes freshly recovered from the ovary (figure 1A and 1B) were about the same as reported for mouse and rat oocytes [11,21]. Furthermore, PDE3 is the predominant PDE activity in the oocyte (figure 1A and 1B), as reported for rodents [11,21]. This result is also consistent with the presence of PDE3A protein detected by Western blotting in porcine oocytes [26]. PDE3A messenger RNA was detected in the oocyte by in situ hybridization in rat and by RT-PCR in mouse and these findings are consistent with the presence of PDE3A mRNA in porcine oocyte (figure 1D) [11,27]. The reported levels of cAMP during IVM range from 2 to 10 fmol [28], whereas the level of cAMP-degrading activity in denuded porcine oocytes reported in this study is around 2.5 fmol of cAMP degraded per minute per oocyte throughout IVM (figure 1B and 2). These results suggest that porcine oocytes replenish their cAMP content approximately every minute. Under our culture conditions, despite the maximum at 13 hours, total and cilostamide-sensitive (PDE3) phosphodiesterase activities were not statistically different during IVM (figure 2). One explanation could be that the timing of GVBD is regulated by multiple cAMP-generating mechanisms. The modulation of cAMP level in the oocyte involves both the permeability of the gap-junctions and adenylyl cyclase activity [1,29]. In addition, the present results do not exclude a possible modulation of PDE3A activity by phosphorylation, which would be difficult to measure because highly synchronized GVBD oocytes would be needed. In the rat, a clear increase of PDE3A activity occurs 30 minutes prior to GVBD [21]. In the pig, the present study shows a peak in cAMP-PDE3 activity 8 hours before GVBD and a loss of the capacity of a PDE3-specific inhibitor to prevent the resumption of meiosis 3 hours before GVBD. Control of oocyte PDE activity has been reported in Xenopus, where microinjection of constitutively active PKB has been shown to increase activity and the GVBD rate [30]. Implication of PKB in the modulation of PDE activity remains a possible mechanism for the resumption of meiosis. From the 6 putative PKA and PKB serine phosphorylation sites in the N-terminus of the murine PDE3A protein sequence (NM_018779.1) described by Shitzukawa and coworkers [11], 5 are present in rat (NM_017337.1), pig (NM_213736.1) and human (NM_000921.3). In the other gene of the same family, PDE3B, at least two of these sites are conserved between rodents and human, and serine-302 is reportedly targeted by PKA and PKB *in vitro *[31,32]. PDE3 has also been reported to be activated by partially purified MAPK and PKB in rat hepatocytes [33]. PKB activation prior to GVBD has never been shown in pig oocytes.

Because cAMP is probably involved in controlling re-entry into the cell cycle in oocytes, the next step was to investigate the role of cAMP in meiotic progression. It was confirmed that OA treatment overcame PDE3-specific inhibition, suggesting that PP1/2A inhibition activates signalling molecules downstream of cAMP-PDE3 in the pig oocyte (figure 5B). This hypothesis is supported by previous reports showing that IBMX-arrested or milrinone (PDE3 inhibitor)-arrested mouse oocytes resume meiosis when PP1/2A is inhibited with OA [25,34]. In the latter study, PDE3 inhibition of OA-treated oocytes delayed GVBD and reduced MPF activation [34]. Interestingly, when using a spontaneous denuded oocyte maturation model, PDE3 inhibition of 3 hours OA-treated oocytes result in higher rates of intermediate stage oocyte compared to OA-treated oocytes alone after 48 hours of IVM (data not shown). These results support our findings, showing that PDE3 inhibition affects meiotic progression in OA-treated oocytes probably by delaying the decrease of MPF activity that allows nuclear maturation to be completed (figures 8 and 9).

In vertebrates, the direct MPF regulators CDC25B and Myt1 are candidate targets for PP1/2A [35]. In fission and budding yeasts, a specific form of PP2A (PP2A-π) directly protects centromeric cohesins from phosphorylation and cleavage, preventing premature sister chromatin separation [36]. The cohesion of centromeres in sister chromatids during metaphase I is of critical importance. In the present study, fifty percent of oocytes remained at the intermediate stage after 36 and 48 hours of IVM (figure 9F), suggesting that inhibition of PP1/2A with OA affects the segregation machinery in a way that impairs homologous chromosome segregation without causing complete meiotic spindle dissolution (figure 6). Similarly, transient PP1/2A inhibition with a relatively high dose of OA (1.24 μM) compromised anaphase initiation and stopped meiosis in metaphase I in mouse oocytes, while low doses (0.124 μM and 0.0124 μM) allowed normal meiosis to continue until metaphase II arrest but with high rates of chromosomal abnormalities [37]. In the present study, the transient OA exposure approach allowed meiosis to proceed to the mature stage. This approach was used successfully in a previous study to reduce the incidence of chromosome segregation defects and to improve the percentage of oocytes reaching metaphase II compared to the continuously OA-treated group [38].

Wang and coworkers have shown that PKA inhibition is one of the earliest events in progesterone-induced resumption of meiosis in Xenopus oocytes [39]. Our results suggest that effective PKA inhibition occurs late after the start of IVM, since stimulation of the cAMP/PKA pathway by PDE3 inhibition after 15 hours of IVM prevents GVBD completely (figure 3A). PDE3 inhibition after 19 hours of IVM has no effect on the completion of meiosis (figure 4). In Xenopus, a similar experiment also demonstrated that reactivating the cAMP/PKA pathway after GVBD has no effect on the completion of meiosis [39]. OA-stimulated oocytes react differently by being delayed at an intermediate stage in the presence of the PDE3 inhibitor (figures 8 and 9). The reason for this is unclear; however, the capacity of the cumulus cells to transfer cAMP to the oocyte could explain the effect. PDE3 inhibition, combined with insufficient cAMP transfer, could prevent intracellular cAMP levels reaching a certain threshold and compromise PKA activation. The level of connexin 43 and its phosphorylation status have been shown to vary in porcine cumulus cells during IVM [40]. Cyclic AMP has also been shown to inhibit proteasomal proteolysis in rat sertoli cells, COS-1 African monkey kidney cells and Y1 mouse adrenocortical tumour cells [41,42]. Interestingly, porcine oocytes stop their nuclear maturation in metaphase I when proteasomal degradation is inhibited [43]. It is possible that PDE3 inhibition in OA-treated oocytes leads to a rise in cAMP level sufficient to prevent proteasomal degradation and consequently delays the transition from intermediate to mature stage. The mechanism by which PDE3 inhibition maintains oocytes in the intermediate stage under OA-treated conditions will require further investigation.

## Conclusion

In summary, the present investigation has revealed new insights into cAMP regulation during the resumption of meiosis in porcine oocytes. It has demonstrated that PDE3A is the major cAMP-degrading PDE activity and regulates meiotic resumption until 3 hours prior to GVBD. It has also demonstrated that PDE3 inhibition affects meiotic progression in OA-treated oocytes. We suggest that cAMP-PDE3A modulates meiotic resumption via a factor located upstream of PP1/2A substrates. This study highlights a distinctive characteristic of porcine oocyte nuclear maturation that makes it a valuable model for studying cAMP modulation during maturation: the very long latency period before the resumption of oocyte meiosis and the late release of the control maintained by the cAMP-PDE3 pathway over prophase arrest. Further experiments will provide a better understanding of the mechanisms involved in the control of PDE3A activity during IVM and the fundamental role of the cAMP/PKA pathway in the molecular mechanisms leading to meiotic maturation.

## Methods

### Chemicals

Unless otherwise notified, all chemicals were purchased from Sigma Chemical Co (St. Louis, MO, USA).

### Ovary collection

Ovaries were collected as previously described [15]. Briefly, ovaries were recovered from a local slaughterhouse, placed in saline (0.9% NaCl) containing antibiotics and antimycotics (100,000 IU/l penicillin G, 100 mg/l streptomycin, 250 μg/l amphotericin B) and maintained at 34°C. Upon arrival in the laboratory, they were rinsed in saline containing antibiotics and antimycotics at 34°C.

### PDE assay

COC were suspended in hypotonic buffer (Tris-HCl 20 mM, pH 7.4, ethylenedioxy-diethylene-dinitrilo-tetraacetic acid (EDTA) 1 mM, ethylene glycol-bis-(2-aminoethyl)-N, N, N', N'-tetraacetic acid (EGTA) 0.2 mM, NaF 50 mM, benzamidine 50 mM, sodium pyrophosphate 10 mM, aprotinin 4 μg/ml, pepstatin 0.7 μg/ml, soybean trypsin inhibitor 10 μg/ml, leupeptin 0.5 μg/ml and phenylmethylsulfonyl fluoride 2 mM) and homogenized by 9 freezing-thawing cycles along with vortex agitation. In all experiments, the hypotonic buffer contained 0.5% Triton X-100. Tris-HCl was purchased from Fisher Scientific Limited (Nepean, ON, Canada). The homogenate was centrifuged for 20 min at 14000 × g to obtain the supernatant. PDE activity was assessed at 34°C in 200 μl final volume with 1 μM cAMP as substrate following the method of Thompson *et al. *[44] with minor modifications [21]; the solution comprised 40 mM Tris-HCl pH 8.0, 10 mM MgCl_2_, 5 mM 2-mercaptoethanol, 0.75 mg/ml BSA (Fraction V), 1 μM cold cAMP and 15 nM [^3^H]cAMP (GE Healthcare, Baie d'Urfé, QC, Canada) (1 × 10^5 ^cpm/tube; 30 Ci/mmol). Measurements were performed in the presence of PDE inhibitors: 3-isobutyl-methylxanthine (IBMX) (1mM, non-specific), cilostamide (Cil) (10 μM, PDE3-specific) and rolipram (Rol) (10 μM, PDE4-specific). Cilostamide and rolipram were purchased from Biomol (Plymouth Meeting, PA, USA). To determine the contributions of different PDEs to the overall PDE activity, the activity measured in the presence of the respective inhibitors was subtracted from the total. Each enzyme assay was performed in triplicate for each experiment and each experiment was repeated at least three times.

### RNA extraction and RT-PCR

RNA extractions were carried out using an "Absolutely RNA Microprep Kit" from Stratagene (La Jolla, CA, USA) according to the manufacturer's protocol. RNA samples were eluted in 15 μl followed by reverse transcription using an "OmniScript RT Kit" from Qiagen (Valencia, CA, USA) and poly (dT) from Ambion (Austin, TX, USA). To each tube, a mixture containing 2 μl of Omniscript 5× Buffer (Qiagen, Mississauga, ON, Canada), 2 μl of 50 μM dNTPs (Qiagen), 0.25 μl of 40 U/μl RNASIN (Promega, Madison, WI, USA) and 1 μl of Omniscript Reverse Transcriptase (Qiagen) was added. The mixture was then incubated at 42°C for 2 hours. The primer pairs were designed on the basis of bovine β-actin (AY141970.1) and human PDE3A sequences (NM_000921.2) and are described in table 1. They were purchased from Integrated DNA Technologies (Skokie, IL, USA). PCR reactions were carried out in a 50 μl reaction volume using Taq polymerase from New England Biolabs (Ipswich, MA, USA). The following cycling conditions were used for all amplifications: 2 minutes 95°C, [1 minute 95°C, 1 minute 60°C, 1 minute 72°C] 35 times, and 10 minutes 72°C. Additional amplifications were performed on an equivalent amount of RNA to exclude genomic DNA contamination. PCR products were visualized by 1% agarose gel electrophoresis and ethidium bromide staining.

**Table 1:** Primers used for PCR reactions. The sequences of the primers used to amplify PDE3A and β-actin mRNA in the oocyte cDNA are shown, along with the expected sizes of the PCR products. Sasseville et al. 2006.

Gene	Primer	Primer sequences	PCR product expected size (bp)
β-actin	β-actin-F	5'-ATCCTGACCCTCAAGTACCCCAT-3'	242
	β-actin-R	5'-TACTCCTGCTTGCTGATCCACAT-3'	
PDE3A	PDE3A-F	5'-GAACAGATGACACTGCTCAAGTT-3'	180
	PDE3A-R	5'-GAGCAAGAATTGGTTTGTCCAG-3'	

### Culture of cumulus-oocyte complexes (COCs) and assessment of meiotic status

Media, COC collection, selection and culture conditions were described previously [15]. Briefly, a group of 30 to 50 COCs were selected and washed three times with HEPES-buffered Tyrode medium containing 0.01% (w/v) polyvinyl alcohol (PVA-HEPES) [45]. They were then cultured in Nunclon™Δ 4-well dishes in 500 μl standard porcine IVM culture medium, namely; BSA-free NCSU 23 medium [46] containing 25 μM beta-mercaptoethanol (Bio Rad), 0.1 mg/ml cysteine, 10% (v/v) filtered porcine follicular fluid and gonadotropin supplements at final concentrations of 2.5 IU/ml hCG (Ayerst Laboratories, Inc., Philadelphia, PA, USA) and 2.5 IU/ml PMSG (Intervet, Whitby, ON, Canada). Wells were covered with a mineral oil overlay. Oocyte nuclear status was assessed using aceto-orcein as previously described [15]. Oocyte nuclear maturation status was classified as (1) GV stage oocyte when displaying a germinal vesicle, (2) intermediate stage oocyte when displaying germinal vesicle breakdown and condensed chromatin or metaphase I organised chromatin, or (3) mature stage oocyte when displaying anaphase I, telophase I or metaphase II DNA configuration.

## Experimental design

### Contributions of different cAMP-degrading PDEs in the oocyte

In experiment 1, cAMP-degrading PDE activity was measured in the COC after recovery from the ovary, and the individual contributions of the PDE3 and PDE4 isoenzymes were determined by the method described above. To evaluate the distribution of PDE activity further, overall PDE activity and specific PDE3 activity were measured in both COC and denuded oocytes. The results are presented as total and cilostamide-sensitive (PDE3) cAMP-PDE activities. The presence of PDE3A mRNA was confirmed in the oocyte by RT-PCR amplification. Total RNA was extracted and reverse transcribed from 100 denuded oocytes. The resulting cDNA was used as template for PDE3A PCR amplification. To verify the absence of contaminating genomic DNA from the RNA extract, PCR was also performed directly on the RNA sample (data not shown). In experiment 2, PDE activity was measured in the oocyte during IVM. After the IVM culture, COC were stripped of their cumulus cells and the denuded oocytes were rinsed in PVA-HEPES and snap-frozen in liquid nitrogen. Cyclic AMP-degrading PDE activity was measured and total, IBMX-sensitive and cilostamide-sensitive PDE activities are presented.

### Time-dependent ability of specific PDE3 inhibition to maintain COC in meiotic arrest

In experiment 3, oocytes were cultured and meiotic maturation was monitored every 6 hours to evaluate the moment of resumption of meiosis. On the basis of previous studies, it is known that inhibiting PDE3 with cilostamide at the beginning of IVM prevents GVBD [15]. To determine the cAMP-sensitive period during which oocytes were not irrevocably committed to resuming meiosis and could still be maintained at the GV stage, addition of cilostamide to the IVM medium was postponed and nuclear status was assessed after 24 hours of IVM. In experiment 4, to determine whether PDE3 inhibition affected meiotic progression, COC were kept in normal IVM conditions for the first 19 hours. Cilostamide was added to half of them and the other half were maintained under normal IVM conditions. The oocytes were denuded and were fixed for assessment of nuclear maturation after 19, 24, 30, 36 and 48 hours of IVM. Oocyte nuclear maturation status was classified as described above.

### Effects of short OA exposure in the presence of PDE3 inhibition on oocyte nuclear maturation

In experiment 5, oocytes were continuously exposed to OA during IVM in order to study the effect of PDE3 inhibition on the resumption of meiosis in premature-GVBD oocytes treated with OA. COC were cultured in OA-supplemented media for 6 and 12 hours and the nuclear status of the oocytes was evaluated. The effect of PDE3-specific inhibition on the resumption of meiosis was assessed by exposing OA-treated oocytes to cilostamide for 24 hours of IVM. The results are reported as mean percentages of arrested, intermediate or mature stage oocytes. In experiment 6, to study the effect of PDE3 inhibition on the progression of meiosis past GVBD, three conditions of transient OA exposure were tested and compared to control conditions: (1) 1 hour OA-exposure followed by 11 or 23 hours of normal IVM; (2) 3 hours OA exposure followed by 9 or 21 hours of normal IVM; and (3) continuous OA exposure for 12 or 24 hours. The percentages of arrested, intermediate and mature stage oocytes, as described for experiment 4, are reported here after 12 hours and 24 hours of IVM. The percentages of oocytes displaying a scattered condensed chromatin configuration were also evaluated and are included in the intermediate stage percentage. Representative pictures of normal metaphase I and OA-treated condensed scattered chromatin oocytes are shown. In experiment 7, to assess the effect of PDE3 inhibition on 3 hours OA-treated COC, the COC were treated with OA for three hours followed either by normal culture conditions or treatment with the PDE3 inhibitor cilostamide. Oocytes were collected after 24 hours of IVM for assessment of nuclear maturation. The percentages of oocytes in arrested, intermediate or mature stages after IVM are reported here. In experiment 8, the effects of PDE3 inhibition on OA-treated COC were studied throughout the IVM period. Oocytes were exposed for 3 hours to OA followed by PDE3 inhibition up to 48 hours. Samples were fixed at 12, 24, 36 or 48 hours for assessment of nuclear status.

### Statistical analyses

Statistical analyses were performed using Prism 4.00 GraphPad for Windows (GraphPad Software, San Diego, CA, USA). The onsets of 50% GVBD and of 50% cilostamide efficacy were calculated using non-linear regression with a sigmoidal dose-response equation. Statistical significances of PDE activity measurements and nuclear maturation status were assessed using one-way ANOVA analysis and the Bonferroni multiple comparison *post-hoc *test to identify individual differences between means. Probabilities of less then 0.05 were considered statistically significant.

## Abbreviations

GV: germinal vesicle; cAMP: 3'5'-cyclic adenosine monophosphate; PKA: cAMP-dependent protein kinase; MPF: M-phase promoting factor; PDE: phosphodiesterase; GVBD: GV breakdown; MAPK: mitogen-activated protein kinase; COC: cumulus-oocyte complex; DO: denuded oocyte; IVM: *in vitro *maturation; IBMX: 3-isobutyl-methylxanthine; OA: okadaic acid; PP1/2A: protein phosphatase 1/2A; Cil: cilostamide; Rol: rolipram.

## Authors' contributions

All authors participated in the design of the study. MS carried out PDE activity measurements and oocyte maturation studies and drafted the manuscript. MS, NC, and CG collected the oocytes. NC and CG performed the PDE assays. FJR conceived the study, participated to its design and helped in the writing of the manuscript. All authors read, modified and approved the final manuscript.
